# The Treatment of Uterine Fibroids

**Published:** 1894-03

**Authors:** 


### THE TREATMENT OF UTERINE FIBROIDS.

Dr. Augustin H. Goelet, in a paper read before the New York County Medical Association, (*American Medico-Surgical Bulletin,* January 1, 1894,) says the important question which arises in dealing with these growths is, When is interference demanded, and what treatment is indicated ? A careful review of the literature of the subject, and the opinions of those who are entitled to be regarded as speaking authoritatively, leads to the conclusion that the majority do not regard the removal of these tumors indicated unless they give rise to sufficient inconvenience to warrant the risk of the operation and the mutilation which it involves ; that the mortality attending their removal is still too great, even in the hands of expert operators, to warrant its being lightly undertaken. If, then, it is possible to relieve the symptoms caused by these growths, the actual necessity for operative interference is narrowed down to a very small field.

The writer does not agree with certain ultra-gynecologists, that these tumors should all be removed when they are small and cause no inconvenience, but he strongly urges that they should be submitted to treatment in this stage, because treatment yields the best results when the tumor is small and of recent growth. The indication for the different methods usually employed are carefully reviewed—electricity, curettement, hysterectomy, and removal of the appendages.

Of electricity, he says that frequently a symptomatic cure is all that may be anticipated. The results that may be obtained by this method are classified as follows:

1. Cure of coëxisting endometritis.
2. Loosening of adhesions between contiguous peritoneal surfaces.
3. Relief of pain and pressure symptoms.
4. Control of hemorrhage.
5. Arrest and some retrogression of the growth.

The writer takes a bold stand in favor of vaginal puncture, believing it to be perfectly safe, if properly done and strict asepsis is observed ; that is, if as much care is taken with this as with other grave surgical operations. He believes that more success would have followed the use of this agent if puncture had not been abandoned as a hazardous measure. He believes, likewise, that it is important to discriminate in the choice of the pole to be employed against growths of different structure, just as important, in fact, as in dealing with such growths as warts, moles and nævi upon the external surface of the body. That is, when the structure is hard and fibrous, the negative pole should be selected, and that when it is soft or myomatous, the positive.

The writer lays stress upon the fact that both subperitoneal and submucous fibroids, when pedunculated, are not amenable to treatment by this agent. He positively declares that, though assertions to the contrary have been made in some quarters, the proper use of electricity in these cases does not complicate a subsequent operation for the removal of the tumor; but, on the contrary, its use facilitates the removal of adhesions and produces a marked improvement in the general condition of the patient. In fact, he often employs it for improving the local condition and for building up the health of the patient preparatory to an operation. It has been asserted that the symptoms return after discontinuing treatment, but the writer believes that, when this occurs, it is either due to a mistaken diagnosis or a faulty technique, which may be unavoidable.

Curettement he thinks is useful as a preliminary measure, but it does not yield a permanent result. In support of this opinion, attention is directed to the fact that the mucous membrane, removed by the curette, is rapidly reproduced and the same causes for the hemorrhage which previously existed still remain. For the control of hemorrhage when both these measures fail, he advocates ligation of the uterine arteries *per vaginam*, as suggested by Martin, of Chicago. He expresses no confidence in ergot alone in these cases, but regards it as a useful auxiliary in the treatment of certain submucous and soft interstitial myomata when it is desirable to excite urine contraction.

Goelet believes that the principal indication for hysterectomy is to be found in large subperitoneal and very large and hard interstitial growths, which yield little, if at all, to any form of treatment. In these cases, even if the symptoms are relieved, their size is usually a source of so much inconvenience as to warrant the risk of their removal. This operation would also be indicated where treatment fails to permanently control the symptoms, when the tumor is situated unfavorably for treatment and when complicated by disease of the appendages.

